# Complete response to FOLFOX4 therapy in a patient with advanced urothelial cancer: a case report

**DOI:** 10.1186/1756-8722-3-4

**Published:** 2010-01-20

**Authors:** Yu Ri Seo, Se Hyung Kim, Hyun Jung Kim, Chan Kyu Kim, Seong Kyu Park, Eun Suk Koh, Dae Sik Hong

**Affiliations:** 1 Division of Hematology & Oncology, Department of Internal Medicine Soonchunhyang University College of Medicine, Bucheon, Korea; 2 Department of PathologySoonchunhyang University College of Medicine, Bucheon, Korea

## Abstract

No standard has been established for salvage therapy in gemcitabine refractory advanced urothelial cancer. We report the complete response to FOLFOX4 therapy of a metastatic urothelial cancer patient, for whom adjuvant gemcitabine plus cisplatin combination chemotherapy had failed. A 54-year-old male patient with urothelial cancer (transitional cell carcinoma) in the right kidney underwent three rounds of adjuvant gemcitabine-cisplatin chemotherapy after extensive radical nephrectomy. However, he had new liver, lung metastases and synchronous two separate primary colon cancer. The lung metastasis lesion was confirmed as a metastatic urothelial cancer via percutaneous transthoracic needle biopsy (PTNB). Liver and lung metastasis lesions disappeared after the 4th cycle of FOLFOX4 chemotherapy. In addition, colon cancer also disappeared after the 8th cycle of FOLFOX4 chemotherapy. The patient was still showing a complete response after 4 months. Clinical trials using the FOLFOX regimen as salvage therapy for gemcitabine-refractory advanced urothelial cancer are warranted.

## Background

Most urothelial cancer develops from the urinary bladder, while urothelial cancer of the upper urinary tract is uncommon, accounting for only 5 to 10% of all renal tumours[1]. The standard therapy for urothelial cancer is surgical resection, although cisplatin-based combination chemotherapy increases the survival in metastatic advanced urothelial cancer [2-4]. Nevertheless, a complete response is very rare, and most patients die within 2 years of diagnosis[5]. At present, the standard therapy is gemcitabine-cisplatin combination therapy because M-VAC (methotrexate, vinblastine, doxorubicin, cisplatin), which was previously the standard therapy, has a mortality due to toxicity exceeding 3% [5-7]. No standard has been established for salvage therapy in gemcitabine-refractory advanced urothelial cancer, and many ongoing clinical trials are examining new agents.

We report a complete response to FOLFOX-4 therapy in a patient with metastatic urothelial cancer who developed lung metastases and an additional primary colon cancer after a radical nephrectomy for urothelial cancer.

## Case presentation

**Figure 1:**
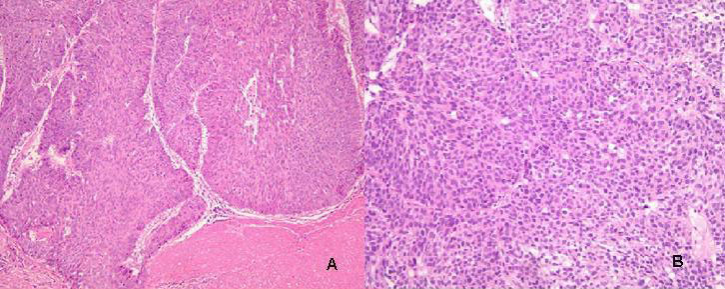
**A: The pelvocalyceal tumor of the kidney reveals high-grade urothelial carcinoma (H&E, ×100)**. **B**: PTNB from lung shows metastatic urothelial carcinoma (H&E, ×200).

A 54-year-old male with urothelial cancer (transitional cell carcinoma) was transferred to the hemato-oncology department after the discovery of lung metastases. Three months previously, he had undergone a radical nephrectomy and hilar lymphadenectomy for a left kidney mass, which was identified as invasive papillary urothelial carcinoma, extending to the renal parenchyma. The resection margin was free from carcinoma, although there was metastatic carcinoma in one out of two lymph nodes (pT3N3 M0) (Figure 1A). No metastatic lesion was found on chest computed tomography (CT) or on abdomen CT before surgery. Postoperatively, he underwent three rounds of adjuvant chemotherapy with gemcitabine (1000 mg/m^2 ^D1, 8, 15) and cisplatin (75 mg/m^2 ^D1).

**Figure 2:**
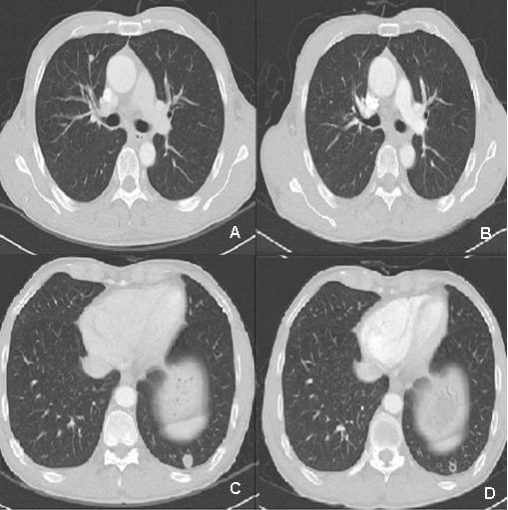
**A, B: Chest CT demonstrating hematogenous metastastatic nodule in RML (arrow, A) disappeared after 4^th ^FOLFOX4 cycles (B)**. **C, D**: Chest CT demonstrating hematogenous metastastatic nodule in LLL (arrow, C) that formed fibrotic cavity after 4^th ^FOLFOX4 cycles (D).

**Figure 3:**
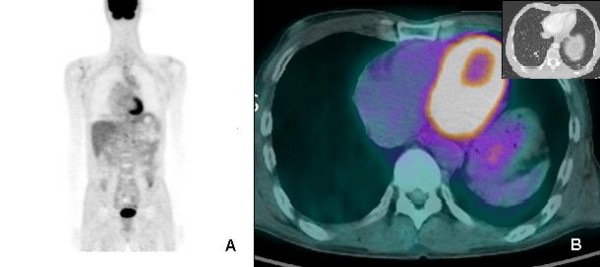
**A: PET CT demonstrating no metastatic lesion after 8^th ^FOLFOX4 cycles**. **B**: PET CT demonstrating no FDG upake in the lung include left lower lobe.

While performing a colonoscopy to investigate hematochezia, a second primary cancer, an adenocarcinoma of the colon, was discovered in the transverse (anal verge 50 cm) and sigmoid (anal verge 20 cm) colon. The level of carcinoembryonic antigen (CEA) was normal, and abdominal CT showed 1.7-cm wall thickening in the sigmoid colon, but no measurable changes in the transverse colon. Moreover, multiple lung metastases were seen on chest CT (Figure 2A, 2C). A lung metastasis was confirmed to be urothelial cancer after a percutaneous transthoracic needle biopsy (Figure 1B) performed on a left lower lobe posterior segment metastatic lesion. The patient underwent FOLFOX-4 (oxaliplatin 85 mg/m^2 ^IV over 2 hours D1; leucovorin 200 mg/m^2 ^over 2 hrs, D1, 2; 5-fluorouracil (5-FU) 400 mg/m^2 ^IV bolus, and 5-FU 600 mg/m^2 ^IV over 22 hrs as a continuous infusion repeated every 2 weeks) for colon cancer and metastatic urothelial cancer, because he refused surgery for the colon cancer. After four rounds of chemotherapy, the lung metastases all disappeared, except one fibrotic cavitary lung lesion (Figure 2B, 2D). There was no hematologic or non-hematologic toxicity other than mild grade 1 nausea, and no delayed treatment schedule. Abdominal and chest CT performed after eight rounds of chemotherapy still showed no metastatic lesions, and positron emission tomography-computed tomography (PET-CT) showed no metastatic lesion (Figure 3A), with no ^18^F- fluoro-2-deoxyglucose (FDG) uptake in the fibrotic cavitary lesion in the lung (Figure 3B). In addition, CR of the colon cancer seen in the transverse and descending colon was also confirmed by colonoscopy and PET-CT after eight rounds of chemotherapy. Nevertheless, regional radiotherapy and rescue chemotherapy are being considered because of enlargement of a left para-aortic lymph node seen on abdominal and chest CT after the twelve rounds of FOLFOX chemotherapy. Therefore complete response was maintained for four months, from after four rounds (11/2008) until twelve rounds (3/2009) of FOLFOX chemotherapy.

## Discussion

For the last 15 years, M-VAC chemotherapy was used to treat metastatic or advanced urothelial cancer, and gave a tumor response of 50~70% with increased survival in 15~20% of patients[2,8,9]. However, the reported mortality related to therapy exceeded 3%, and 25% of the patients developed neutropenic sepsis, so its use was limited to young patients or those with good general performance[10]. Gemcitabine was reported to give a good response in urothelial cancer and has low toxicity[7]. Finally, a phase III study of gemcitabine-cisplatin (G-C) showed a similar response rate and survival compared with M-VAC, but lower toxicity and better safety. Consequently, G-C is now used widely to treat urothelial cancer[5]. Unfortunately, the tumor recurs in most patients within one year[9,10], necessitating secondary therapy after the failure of standard therapy. Although many ongoing clinical trials are examining this, no treatment has been established as secondary therapy after failure of G-C or M-VAC chemotherapy.

Oxaliplatin is more potent than cisplatin *in vitro *and has shown efficacy in preclinical studies against many tumor cell lines[11,12]. It has also proved efficacious in several phase II trials and is considered less nephrotoxic than cisplatin and causes less bone marrow suppression than carboplatin[10,13,14]. However, the activity of an oxaliplatin single regimen for urothelial cell cancer was minimal in phase II studies by Moore *et al*.[13] and Winquist *et al*.[14]. Therefore, we suggest that our case of TCC showed a complete response due to synergistic effects of FOLFOX-4, rather than to those of oxaliplatin as a single drug. The efficacy of 5-FU and leucovorin combination therapy for colorectal cancer is widely known[15,16]. The efficacy of 5-FU in advanced urothelial cancer is unclear, but a review of published studies in 1987 described response rates of about 15% using unmodulated single agent 5-FU[17]. In combination with alpha interferon, a partial response rate of 30% was obtained[18]. Recently, a phase II trial of continuous 5-FU infusion showed a median progression-free survival of 1.9 months and a median overall survival of 6.5 months[19].

The FOLFOX regimen, which is a combination of 5-FU, leucovorin, and oxaliplatin, can involve various doses and schedules. It shows low toxicity and good efficacy for colon cancer and stomach cancer, so it is used widely at present. The addition of new agents such as bevacizumab is expected to increase the complete response and survival rates for patients with metastatic colorectal cancer [20,21]. There are few reports of FOLFOX therapy for urothelial cancer, only a phase II trial by Lorenzo *et al*., published in 2004. They used FOLFOX-4 in 18 patients who had previously been treated for urothelial cancer, and reported only low-grade toxicity and a 19% overall response rate, all partial responses[22].

Our patient was given FOLFOX therapy because the urothelial cancer failed to respond to G-C combination therapy, as metastases were discovered and there was an accompanying second primary colon cancer. He showed a complete response in both the metastatic urothelial cancer and colon cancer. In addition to the ongoing clinical studies of gallium nitrate, ifosfamide, pemetrexed, vinflunine, and molecular targeting agents, a clinical trial of FOLFOX-4 therapy for urothelial cancer seems to be warranted[23].

## Consent

Written informed consent was obtained from the patient for publication of this case report and accompanying images. A copy of the written consent is available for review by the Editor-in Chief of this journal.

## Competing interests

The authors declare that they have no competing interests.

## Authors' contributions

SYR was responsible of the acquisition of data, drafting the manuscrips; KHJ was responsible of the clinical management of the patient, scientific revision, discussion and editing of the manuscript; KSH, KCK, PSK were involved in clinical management of the patient and interpretation of data; KES was responsible of the interpretation of pathology; HDS was supervisor of clinical management of the patient and interpretation of data.
